# Exploring Therapeutic Avenues in Lung Cancer: The Epigenetic Perspective

**DOI:** 10.3390/cancers15225394

**Published:** 2023-11-13

**Authors:** Raluca Munteanu, Ciprian Tomuleasa, Cristina-Adela Iuga, Diana Gulei, Tudor Eliade Ciuleanu

**Affiliations:** 1 Medfuture Research Center for Advanced Medicine, Iuliu Hatieganu University of Medicine and Pharmacy, 400347 Cluj-Napoca, Romania; muresan.raluca.andrada@gmail.com (R.M.); ciprian.tomuleasa@umfcluj.ro (C.T.); 2 Academy of Romanian Scientists, Ilfov 3, 050044 Bucharest, Romania; 3 Department of Hematology, Iuliu Hatieganu University of Medicine and Pharmacy, 400012 Cluj-Napoca, Romania; 4 Department of Hematology, Ion Chiricuta Clinical Cancer Center, 400124 Cluj-Napoca, Romania; 5 Department of Proteomics and Metabolomics, Research Center for Advanced Medicine–MEDFUTURE, “Iuliu Hatieganu” University of Medicine and Pharmacy Cluj-Napoca, Louis Pasteur Street 6, 400349 Cluj-Napoca, Romania; iugac@umfcluj.ro; 6 Department of Pharmaceutical Analysis, Faculty of Pharmacy, “Iuliu Hatieganu” University of Medicine and Pharmacy, Louis Pasteur Street 6, 400349 Cluj-Napoca, Romania; 7 Department of Oncology, Iuliu Hatieganu University of Medicine and Pharmacy, 400012 Cluj-Napoca, Romania; 8 Department of Oncology, Prof. Dr. Ion Chiricuta Oncology Institute, 400015 Cluj-Napoca, Romania

**Keywords:** epigenetics, DNA methylation, histone modifications, lung cancer

## Abstract

**Simple Summary:**

Understanding epigenetic processes could revolutionize lung cancer care, enhancing diagnosis, prognosis, and treatment. Promising clinical trials, featuring various drug treatments and immunotherapy, offer optimism for patients with lung cancer, aiming for extended, better-quality lives with reduced side effects. However, comprehending how epidrugs function in these treatments remains a crucial challenge. Bridging this knowledge gap will enhance our ability to use epidrugs effectively, ultimately benefiting lung cancer patients by improving their treatment outcomes and quality of life.

**Abstract:**

Lung cancer, primarily non-small cell lung carcinoma (NSCLC) and small cell lung carcinoma (SCLC), is distinguished by its high prevalence and marked mortality rates. Traditional therapeutic approaches, encompassing chemotherapy, radiation, and targeted therapies, frequently show limited efficacy due to acquired resistance and notable side effects. The objective of this review is to introduce a fresh perspective on the therapeutic strategies for lung cancer, emphasizing interventions targeting the epigenetic alterations often seen in this malignancy. This review presents the most recent advancements in the field, focusing on both past and current clinical trials related to the modulation of methylation patterns using diverse molecular agents. Furthermore, an in-depth analysis of the challenges and advantages of these methylation-modifying drugs will be provided, assessing their efficacy as individual treatments and their potential for synergy when integrated with prevailing therapeutic regimens.

## 1. Introduction

Lung carcinoma is a prevalent malignancy associated with a high incidence of both morbidity and mortality, ranking prominently among the leading contributors to cancer-related fatalities [1]. The elevated mortality rate can be largely attributed to the frequent diagnosis of the disease at advanced stages, which limits the prospects for effective treatment and results in a low five-year overall survival rate of 18.5% [2].

Tobacco use accounts for approximately 80–90% of lung carcinoma cases, with smoking representing the primary risk factor for its development [3]. Histologically, lung carcinoma is classified into two primary histological subtypes: non-small cell lung carcinoma (NSCLC), constituting approximately 85% of cases, and small cell lung carcinoma (SCLC), making up the remaining 15% [4].

Carcinomas, including lung carcinoma, exhibit substantial genetic, transcriptional, and phenotypic diversity, significantly influencing disease progression, metastasis, and therapeutic responsiveness [5]. Within carcinomas, intertumoral heterogeneity, not solely driven by genetic mutations, prominently stems from epigenetic alterations, which often surpass somatic mutations in prevalence [6].

Epigenetics, characterized by heritable and reversible gene expression changes without altering DNA sequences, plays a pivotal role in the dysregulation of oncogenes and tumor suppressor genes, thereby fostering lung cancer development [7]. Specifically, DNA methylation patterns undergo modifications, particularly in lung cancer subtypes like NSCLC and SCLC. Genes such as *p16INK4a*, *p15INK4b*, *RASSF1A*, and *MGMT* exhibit heightened promoter methylation in tumors from smokers [8,9]. Additionally, certain cancer cells display deficient DNA methyltransferase (DNMT) activity, implying an important underlying role of DNA methylation in cancer.

Nevertheless, the role of DNA methylation in cancer is intricate, and the effects of targeting it with methyltransferase inhibitors vary depending on cancer type and stage. Histone modifications, including acetylation, methylation, and phosphorylation, impact chromatin structure and gene expression regulation. The overexpression of histone deacetylases (HDACs) in lung cancer has spurred investigations into HDAC inhibitors as potential therapeutic agents [10].

Epigenetic therapy has emerged as a promising strategy in lung cancer treatment. DNMT inhibitors like azacytidine and decitabine exhibit the potential to reactivate silenced tumor suppressor genes by demethylating hypermethylated regions. Additionally, HDAC inhibitors, disrupting cancer cell processes and gene expression, are under scrutiny for their antiproliferative effects in NSCLC [11].

Several HDAC inhibitors (HDACi), including SL142, SL325 [12], HTPB, and CG0006 [13], have shown promise in inducing apoptosis and inhibiting the proliferation of NSCLC cells.

Moreover, HDAC inhibition has been observed to reduce the responsiveness of tumor cells to TNF-alpha-mediated activation of the NF-kappa B pathway. Despite limited single-agent efficacy, few objective responses, and substantial toxicity observed in clinical trials, HDAC inhibitors have demonstrated potential in pre-clinical studies for the treatment of NSCLC [14,15]. To address these challenges and enhance therapeutic outcomes, combination strategies involving the synergy of HDAC inhibitors with other treatments have been explored. One such combination approach, investigated in a phase II clinical trial, involved the combination of the HDAC inhibitor Vorinostat with Paclitaxel and Carboplatin in advanced lung cancer patients. This study revealed that the addition of Vorinostat improved treatment outcomes [16]. However, a subsequent phase III study of the same combination was terminated due to insufficient effectiveness (ClinicalTrials.gov identifier NCT00473889).

Combining epigenetic therapies with conventional treatments has shown promise in sensitizing cancer cells to chemotherapy and radiation therapy. Understanding the epigenetic landscape of lung cancer, encompassing DNA methylation, histone modifications, and gene expression interplay, offers valuable insights for tailored epigenetic therapies. Utilizing the potential of epigenetic therapies alongside conventional treatments presents a hopeful avenue for enhancing the effectiveness of chemotherapy and radiation therapy in treating lung cancer, underscored by a deeper comprehension of the epigenetic dynamics [17].

## 2. Overview of Lung Cancer Epigenetics

Oncogenesis is a complex phenomenon that is dependent on multiple factors, especially genetic, epigenetic, and environmental factors [18]. Advances in the field of epigenetics have provided a more comprehensive understanding of the carcinogenesis mechanism. Specifically, DNA hypermethylation is a hallmark of lung cancer and an early event in carcinogenesis [19]. Also, the roles of non-coding RNAs (ncRNAs) in RNA–RNA interactions, epigenetic and posttranscriptional regulation, and other biological processes are increasingly being recognized [20]. When these epigenetic factors are altered, important oncogenes and tumor suppressor genes are dysregulated to the advantage of malignant progression [21].

Cancer hallmarks such as proliferation, invasion, metastasis, apoptosis, and cell cycle regulation are all influenced by epigenetic changes in lung cancer. Epigenetic alterations in lung cancer can lead to dysregulation of major signaling pathways, including MAPK/ERK and PI3K/AKT, and NOTCH, resulting in enhanced cell growth, survival, and resistance to cell death. These changes contribute to the characteristic features of lung cancer [22,23]. Within these pathways, specific genes can undergo methylation or demethylation, contributing to the hallmark features of lung cancer. For instance, tumor suppressor genes like *RASSF1A* [24] and *DUSP4* [25] within the MAPK/ERK pathway may be subjected to hypermethylation and subsequent silencing, promoting tumorigenesis. In contrast, key tumor suppressors like *PTEN* in the PI3K/AKT pathway can become hypermethylated and functionally inactivated [26]. Simultaneously, demethylation events may lead to the activation of oncogenes, fostering cell growth and survival [27]. Moreover, epigenetic modifications may disrupt NOTCH signaling, with genes like *NOTCH1* undergoing methylation-induced silencing. On the flip side, demethylation of certain genes can lead to overactive NOTCH signaling, influencing cell proliferation and differentiation [28].

Concurrently, epigenetic processes shed light on the identification of possible cancer biomarkers for use in diagnostics, follow-up, prognosis, risk assessment, and oncotherapy [29].

## 3. Aberrant DNA Methylation and Gene Modification in Lung Cancer

DNA methylation is one of the most extensively studied epigenetic events in the mammalian genome [30]. It involves the addition of a methyl group (CH3) to a DNA molecule, which can alter gene expression without changing the underlying DNA sequence. This epigenetic change, being one of the main mechanisms controlling the transcriptional battery of the cell [8], is linked to various cancer-related processes, including gene silencing, chromosomal instability, and altered cell proliferation [31].

Aberrant DNA methylation is a common feature of NSCLC, with hypermethylation of tumor suppressor genes and hypomethylation of oncogenes being commonly observed [32]. One of the best-characterized examples of hypermethylation in NSCLC is the promoter methylation of the tumor suppressor gene *P16INK4a*, which leads to its silencing and contributes to cell proliferation [33]. Other relevant examples of genes commonly hypermethylated in lung cancer are described in Table 1.

As a therapeutic approach, recent studies have suggested that increasing DNA methylation using methyltransferase inhibitors may have potential benefits in certain types of cancer, despite the inhibition of DNMT enzymes being a common approach for cancer treatment [34]. Hypermethylation of tumor suppressor genes and silencing in NSCLC influence various biological processes, including cell cycle control (p16), DNA repair (*MGMT*), apoptosis (*DAPK*), RAS (*RASSF1A*) and Wnt (*APC*) signaling, and invasion suppression (*CDH13* and *TIMP3*) (Table 1). Candidate gene-based studies have shown that hypermethylation of tumor suppressor genes, including *p16, MGMT, RASSF1A*, and *APC*, occurs early in neoplasia and rises with carcinoma development, implicating these processes in disease onset and progression [35].

**Table 1:** Representative genes that are often hypermethylated in lung cancer.

Gene	Epigenetic Modifications	Reported Histological Type	Mechanism	References
*RASSF1A*	hypermethylation	NSCLC, SCLC	Hypermethylation of the *RASSF1A* gene promoter region in lung cancer disrupts *RASSF1A*’s tumor suppressor function, potentially leading to dysregulated cell cycle progression and resistance to Ras-induced apoptosis.	[36]
*SEMA3B*	hypermethylation	NSCLC, SCLC	Hypermethylation of *SEMA3B* gene promoter region results in decreased *SEMA3B* expression, which can compromise its tumor-suppressive functions, promote tumor growth, and potentially contribute to metastasis.	[37]
*DAPK*	hypermethylation	NSCLC	Cell cycle progression, apoptosis, cell migration.	[38]
*P14*	hypermethylation	NSCLC	Hypermethylated *P14* gene results in increased activity of CDK4 and CDK6, leading to uncontrolled cell cycle progression and excessive cell growth.	[39]
*FHIT*	hypermethylation	NSCLC	*FHIT* hypermethylation induces inactivation of *FHIT* gene, facilitating genomic instability, promoting clonal expansion and enhancing survival under selective pressures.	[40,41]
*PTEN*	hypermethylation	NSCLC	Inhibitor of the AKT/MTOR pathway and cell cycle.	[42]
*CDKN2A*	hypermethylation	NSCLC	Hypermethylation of *CDKN2A* contributes to tumorigenesis through the inactivation of tumor suppressor genes, specifically *p16INK4a* and *p14ARF.*	[43]
*CDH1*	hypermethylation	NSCLC	Facilitates cellular adhesion while restraining cellular motility, invasion, and metastasis.	[44]
*CDH13*	hypermethylation	NSCLC	Loss of E-cadherin weakens cell–cell adhesion, promoting cancer cells’ ability to detach from the primary tumor and invade surrounding tissues.	[45]
*BLU*	hypermethylation	NSCLC	Transcription regulation.	[46]
*TBX-2*	hypermethylation	NSCLC	Hypermethylation of the *TBX-2* promoter region creates a repressive chromatin state that inhibits transcription factor binding and RNA polymerase activity, consequently silencing *TBX-2* gene expression and disrupting its role in transcription regulation, which contributes to tumorigenesis.	[47]
*WIF1*	hypermethylation	NSCLC	Hypermethylation of *WIF1* gene promoter region silences *WIF1* expression by adding methyl groups to CpG islands, impairing its tumor-suppressive function and promoting tumor growth and progression.	[48]

The gene *P16INK4a*, also known as cyclin-dependent kinase inhibitor 2A (*CDKN2A*), is a critical tumor suppressor gene. It controls the cell cycle by inhibiting the activity of cyclin-dependent kinases (CDKs), particularly CDK4 and CDK6 [49]. By doing so, *p16INK4a* regulates the G1 phase of the cell cycle, preventing uncontrolled cell division. When *p16INK4a* is altered through hypermethylation, mutation, or deletion, the result can be a disruption of the regulatory mechanism, potentially contributing to uncontrolled cell proliferation [43].

In addition to *p16INK4a*, the co-encoded gene *p14ARF* (another product of CDKN2A) is also frequently inactivated in NSCLC [50]. *P14ARF* plays a complementary role in tumor suppression by promoting the stabilization of p53, a crucial protein involved in DNA repair and apoptosis. Therefore, alterations in both *p16INK4a* and *p14ARF* can collectively contribute to the development of NSCLC [51].

A substantial number of cases of NSCLC and SCLC cases exhibit deletions or hypermethylation of the *RASSF1A* gene, ranging from 30% to 40% in NSCLC and as high as from 70% to 100% in SCLC. Additionally, *FHIT* deletions (Fragile Histidine Triad Diadenosine Triphosphatase) or hypermethylation are observed in from 40% to 70% of NSCLC cases and from 50% to 80% of SCLC cases, underscoring their significance in lung cancer progression. Furthermore, *TSLC1* hypermethylation is also a prevalent epigenetic alteration, occurring in over 80% of NSCLC cases [52].

NSCLC tumors from smokers exhibit more promoter methylation in *p16INK4a*, *p151NK4b* [53], *RASSF1A, MGMT, MTHFR*, and *FHIT* genes [54]. Also, in a meta-analysis, Tao Huang et al. revealed significant associations between smoking behavior in NSCLC patients and the hypermethylation of seven genes: *CDKN2A, RASSF1, MGMT, RARB, DAPK, WIF1*, and *FHIT* [55].

In a study led by Steven A. Belinsky and colleagues, an analysis of methylation patterns in 26 genes was conducted in primary lung adenocarcinoma on 175 patients among current, former, and never smokers. Among these genes, 25 displayed methylation, notably, methylation of *TNFRSF10C, BHLHB5*, and *BOLL* was more frequently observed in patients with adenocarcinomas from non-smokers than in those from smokers [56].

It is also important to acknowledge the substantial correlation between hypomethylation in non-small cell lung cancer (NSCLC) and genomic instability. This correlation can result in the activation of oncogenes and the loss of DNA imprinting. For example, in various human cancers, including lung cancer, *IGF2* can undergo loss of imprinting (LOI), causing the silenced maternal allele to reactivate and boost *IGF2* expression. This dysregulation is associated with cancer development, where LOI of *IGF2* has been observed [57].

Within the domain of lung cancer, hypomethylation primarily impacts distinct genomic components, including nuclear elements, LTR elements, segmental duplicates, and subtelomeric regions. In contrast, non-repetitive sequences exhibit a lower occurrence of methylation loss [58].

Moreover, it is important to highlight that several distinct genes demonstrate hypomethylation, such as *MAGE-A3/6* [59] and *BORIS* [60]. The overexpression of *MAGE*, which is associated with the loss of methylation, has been seen in a significant proportion, roughly 75–80%, of cases of non-small cell lung cancer (NSCLC). This highlights the complex relationship between hypermethylation and hypomethylation within the framework of lung cancer [61].

## 4. Epigenetic Therapy in Lung Cancer

### DNMT Inhibitors

The field of epigenetics has opened up new therapeutic possibilities for lung cancer due to the involvement of epigenetic modifications in the development of resistance to targeted treatments, chemotherapy, and radiation. The exploration of the complex epigenetic network that regulates gene expression in cancer cells and the surrounding tumor microenvironment presents a promising approach for therapeutic intervention, thanks to its ability to influence several pathways and address the challenges posed by both inter- and intratumoral heterogeneity [62,63].

Epigenetic therapy encompasses a broad spectrum of modifications, including the demethylation of tumor suppressor genes and the reversal of hypermethylation of oncogenes, all aimed at restoring normal gene expression patterns and inhibiting tumor growth [64].

**Figure 1:**
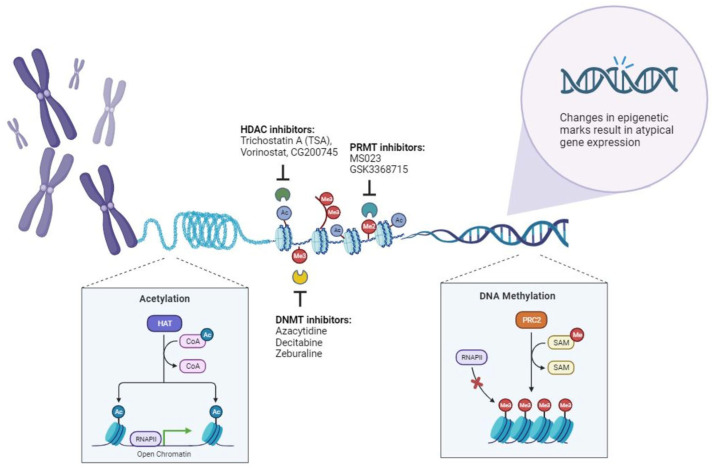
The dynamic of epigenetics, showcasing its mechanisms and relevance in the context of lung cancer. Epigenetic processes are both heritable and reversible, with the key players encompassing DNA methylation, histone modifications, and chromatin organization. Among these mechanisms, post-translational modifications take center stage, encompassing the covalent methylation (Me) and acetylation (Ac) of histone tails, as well as DNA methylation.

In response to this need for developing new therapies, researchers have been actively studying compounds that can modify DNA methylation patterns. The mechanism of action of DNMTi involves the inhibition of DNA methyltransferases, leading to the demethylation of hypermethylated genes, including tumor suppressor genes. This results in the reactivation of these genes and the restoration of normal cellular processes, which can halt the progression of cancer and other diseases [65]. In fact, the Food and Drug Administration (FDA) has authorized histone deacetylase inhibitors (HDACi), DNA methyltransferase inhibitors (DNMTi), and Janus kinase 2 inhibitors as therapies for chromatin regulating therapies [7]. Among DNMT inhibitors (DNMTi), azacytidine and decitabine stand out, demonstrating significant promise in both preclinical and clinical studies for treating cancer and other diseases (Figure 1) [34].

The therapeutic combination of HDACi and DNMTi reduces Myc levels and reverses immunological evasion in lung cancer by accelerating T cell attraction. Specifically, this therapeutic regimen drives the accumulation of CD8+ T cells at the tumor site, even probably induced by the simultaneous elevated CCL5 protein secretion at the bronchoalveolar segment. High levels of CCL5 were previously associated with a longer overall survival in patients diagnosed with NSCLC [66]. Considering the direct antiproliferative effect, but also the immunological implications of the two-compound-based epigenetic therapy, the study proposes the combination of HDACi and DNMTi with immune checkpoint blockade for treatments of patients with lung cancer [67].

In addition to DNMT inhibitors like azacytidine and decitabine, zebularine has emerged as a promising nucleoside DNMT inhibitor and has demonstrated effectiveness in vitro and is considered a promising drug for potential clinical trials [68]. By creating strong covalent bonds between DNMT proteins and DNA that contains zebularine, this substance inhibits DNMT activity [69]. Zebularine’s stability at both acidic and neutral pH levels, unlike azacytidine and decitabine, is crucial for maintaining long-term DNMT inhibition, and its suitability for regular dosing or continuous intravenous infusion ensures consistent and sustained epigenetic therapy, which is vital for effectively countering lung cancer progression [70]. Furthermore, zebularine has the potential to be used in conjunction with other therapeutic interventions such as chemotherapy, immunotherapy, or radiation in the future; therefore, it is a potential medication candidate [71,72].

The main DNMT inhibitors tested in clinical trials for the treatment of lung cancer are presented in Table 2. The studies were selected by performing a search protocol based on the following keywords: “lung cancer” for condition or disease and “DNMT” for other terms on clinicaltrials.gov.

## 5. Histone Alterations

Histones, basic proteins abundant in lysine and arginine residues, are found in eukaryotic cell nuclei. They function as spools around which DNA winds structural units known as nucleosomes. These nucleosomes are then bundled into 30-nanometer strands, which create densely packed chromatin. The role of histones is to prevent DNA from becoming twisted and shield it from damage. Additionally, histones have critical functions in gene regulation and DNA replication [73].

Chemical processes such as acetylation, methylation, phosphorylation, ubiquitination, and sumoylation can modify histone proteins. These changes can alter the connections between the histones and DNA, resulting in changes to chromatin structure and gene expression. Enzymes called histone deacetylases (HDACs) can remove acetyl groups from histones, leading to a more compact chromatin structure that represses gene transcription [74].

### 5.1. HDAC Inhibitors

HDACs were found at high expression in lung cancer [31]. Studies have shown that HDAC inhibitors may have antiproliferative effects in NSCLC, and they are currently being investigated as potential therapeutic options in clinical trials [7]. HDACi disrupts cancer cell processes such as cell proliferation, angiogenesis, differentiation, and apoptosis, causing cell cytotoxicity by influencing the expression of multiple genes and proteins [75]. Typically, HDACi compounds feature a zinc-binding domain connected to a capping group via a straight chain link [76].

HDACi are classified into various subgroups based on their chemical structure, including aliphatic acids (e.g., Sodium butyrate, Phenylbutyrate, and Valproic acid), Benzamides (including Entinostat and Mocetinostat), cyclic peptides (such as Largazole and Romidepsin), and hydroxamic acids (e.g., Trichostatin A (TSA) and Vorinostat/Suberoylanilide hydroxamic acid) [77]. Additionally, some dietary phytochemicals, like sulforaphane and phenethyl isothiocyanate, have been found to suppress HDAC activity, suggesting potential anti-tumor effects [78].

However, targeting HDACs is complex given their various subclasses, some of which have unclear roles and mechanisms of action [76]. Furthermore, HDACs’ enzymatic activity extends beyond histones to several other proteins [79]. Regarding their effectiveness, HDACi like Trichostatin A (TSA), Vorinostat, and CG200745 have exhibited promising results in inhibiting NSCLC cell proliferation [80]. These inhibitors trigger various pathways, including intrinsic mitochondrial and extrinsic/Fas/FasL system death pathways, upregulate cyclin-dependent kinase inhibitor p21, and inhibit *Notch1* signaling [81]. Other HDAC inhibitors (HDACi), including SL142, SL325 [82], HTPB [83], and CG0006 [13], have shown promise in inducing apoptosis and inhibiting the proliferation of NSCLC cells.

Moreover, HDAC inhibition has been observed to reduce the responsiveness of tumor cells to TNF-alpha-mediated activation of the NF-kappa B pathway [84].

Despite limited single-agent efficacy, few objective responses, and substantial toxicity observed in clinical trials, HDAC inhibitors have demonstrated potential in pre-clinical studies for the treatment of NSCLC [14,15]. To address these challenges and enhance therapeutic outcomes, combination strategies involving the synergy of HDAC inhibitors with other treatments have been explored. One such combination approach, investigated in a phase II clinical trial, involved the combination of the HDAC inhibitor Vorinostat with Paclitaxel and Carboplatin in advanced lung cancer patients. This study revealed that the addition of Vorinostat improved treatment outcomes [16]. However, a subsequent phase III study of the same combination was terminated due to insufficient effectiveness (ClinicalTrials.gov identifier: NCT00473889). Another combination strategy involves the use of epigenetic therapy, specifically combining a low dose of the DNA methylation inhibitor azacytidine (CC-486) (40 mg/m^2^/day) with the histone deacetylase inhibitor Entinostat (7 mg fixed dose). In a phase II trial, this combination showed potential in improving the survival of advanced NSCLC patients. However, this approach can cause common side effects like injection site reactions, gastrointestinal disturbances, hyperglycemia, and hematological side effects [16].

Additionally, in vitro studies suggest that 5-aza-2′deoxycytidine can reduce DNA methylation, making lung cancer cells more sensitive to the EGFR inhibitor Gefitinib [85].

The main HDACi tested in clinical trials for treatment of lung cancer are presented in Table 3. The studies were selected by performing a search protocol based on the keywords: “lung cancer” for condition or disease and “HDAC inhibitor” for other terms on clinicaltrials.gov. The search resulted in 76 studies involving different combination therapies. To present the most significant results up to the present day, we selected only the phase 3 studies to be described in more detail.

### 5.2. PRMT Inhibitors and MTAP Deletion in Lung Cancer

Protein arginine methyltransferases (PRMTs), consisting of nine identified enzymes, facilitate arginine methylation, a widespread post-translational modification integral for numerous proteins including histones, transcription factors, and DNA damage repair proteins [86,87]. PRMTs are classified into three distinct types, each contributing uniquely to epigenetic regulation, cell cycle progression, and DNA repair mechanisms. Notably, cancer cells tend to exhibit PRMT dysregulation, particularly through overexpression, leading to altered methylarginine patterns. This makes PRMTs appealing targets for therapeutic intervention [88]. A common PRMT dysregulation, specifically of PRMT5, has been correlated with the loss of MTAP (5-methylthioadenosine phosphorylase) expression in lung cancer, resulting in the accumulation of methylthioadenosine (MTA), which significantly inhibits PRMT5 activity [89,90]. The loss of MTAP expression, as a result of deletion, stands out as a significant area of interest due to its prevalence in over 10% of diverse human malignancies, including in NSCLC [91]. To comprehend the full impact of MTAP loss, it is crucial to examine its occurrence and distribution within specific types of NSCLC. A comprehensive study involving 50 primary NSCLC tissue samples determined that the rate of MTAP deletion stands at approximately 38% of the samples analyzed. The investigation provided insight into the distribution of MTAP deletion across diverse histological subtypes, revealing 50% adenocarcinomas, 42% squamous cell carcinomas, and 8% large cell carcinomas, from the set of samples analyzed. Notably, adenocarcinomas exhibited a heightened incidence of MTAP deletion at 44%, compared to a 29% deletion rate in squamous cell carcinomas [92]. Inhibiting a specific type of PRMT (Type I) has shown promise in treating cells without MTAP. Compounds like MS023 and GSK3368715 have been able to reduce the survival of these cells, pointing towards a new way to target this type of cancer [93,94].

Recent advances in functional genomics elucidated the complex interplay between MTAP and MAT2A, a S-adenosylmethionine (SAM)-producing enzyme, revealing a synthetic lethal interaction. In studies conducted on HCT116 MTAP isogenic pairs, MAT2A emerged as a top hit, underscoring its critical role in this context. Importantly, when MAT2A was knocked down in MTAP−/− cells, a substantial reduction in symmetric arginine dimethylation marks (SDMA) was observed, while MTAP wild-type cells remained unaffected. This highlights a vulnerability in MTAP-deficient cells that could potentially be exploited for therapeutic gains [95]. Additionally, the disruption of MTAP and MAT2A’s interaction is not the only pathway altered in these cells; PRMTs also play a pivotal role in this complex molecular landscape.

Furthermore, Omar Alhalabi et al. conducted an analysis on a cohort of lung adenocarcinoma patients, observing a statistically significant increase in response rates to frontline platinum-based chemotherapy regimens, including pemetrexed, in subjects with reduced CDKN2A/MTAP expression, indicative of 9p21 loss. Conversely, patients with presumed intact 9p21 exhibited lower response rates. The findings from this study provide strong evidence supporting the notion that MTAP deficiency in neoplastic tissues confers a metabolic susceptibility to antifolate agents, notably pemetrexed [96].

An analysis of a lung adenocarcinoma retrospective cohort (*n* = 72) from the BATTLE2 clinical trial (ClinicalTrials.gov ID: NCT01248247) revealed that MTAP deficiency correlates with a heightened response rate to pemetrexed. Another study, conducted by Jing et al., conducted a retrospective analysis of 165 cases of advanced NSCLC undergoing platinum-based chemotherapy and bevacizumab treatment, utilizing IHC to differentiate between MTAP-low and MTAP-high patients. Their findings revealed that the MTAP-low group experienced shorter progression-free survival (PFS), establishing a negative correlation between low MTAP status and survival in advanced LUAD (HR 1.36, *p* = 0.038). Furthermore, the study highlighted methionine adenosyltransferases, MAT1a and MAT2a, as emerging targets of interest due to their critical roles in cell growth and survival and their involvement in the polyamine biosynthesis cycle. In vivo experiments demonstrated that knocking down MAT2a diminished the growth of MTAP-deficient tumor cells. At present, a phase I clinical trial is investigating the potential of IDE397, an inhibitor of MAT2A, in treating advanced solid tumors characterized by the absence of MTAP [97]. These findings underscore the imperative need for prospective clinical trials focusing on MTAP-deficient NSCLC (ClinicalTrials.gov ID: NCT04794699).

Given the histological diversity within MTAP-loss NSCLC, the potential of PRMT5 and MAT2A inhibitors for future treatment, regardless of disease stage, validates the need for further evaluation of MTAP loss as a pathway to innovative therapies in NSCLC.

## 6. Involvement of Epigenetic Modifications in the Development of Lung Cancer Drug Resistance

Therapeutic resistance of cancer cells is a complex mechanism that mainly involves the tumor-favorable modification of the drug targets. Although the chemotherapeutic regimens are now more advanced in action, they are still minimally efficient for advanced forms of cancer. Specifically, it is estimated that in 90% of the cases, chemotherapy inefficacy is based on the activation of drug resistance mechanisms [98].

Important factors that affect medication resistance include the total number of tumor cells or mass present in the patient’s body, also known as the tumor burden or tumor load, determined by adding the largest diameters of all detectable lesions [99]. Also important is the ratio of the slope of tumor growth prior to treatment to the slope of tumor growth following treatment between the nadir and disease progression, as computed for each patient, known as tumor growth kinetics [100]. Furthermore, tumor heterogeneity is essential and includes the various phenotypic characteristics of tumor cells, like shape, gene expression, metabolism, motility, proliferation, and ability to metastasize, as well as their interactions with the immune system, the TME (tumor microenvironment), drugs, and other factors. The resistance mechanism is also influenced by cell type, background mutational load, and the subsequent appearance and selection of new drivers [101].

In terms of specific mechanisms, the drug-resistant phenotype of cancer cells can be caused by inhibition of cell death (apoptosis suppression), drug inactivation, muti-drug resistance, modifications of the drug metabolism, gene amplification, DNA repair, and epigenetic modifications [98].

Given that tumors may react to treatment initially, but because not all neoplastic cells are eradicated, the tiny population, or rare residual drug-resistant cells harboring certain alterations, can seed cancer resurgence. Resistance may or may not include genetic alterations. Some cancer cells may be able to withstand chemotherapy by making use of nutrients in their tumor microenvironment. As a result, TME-related characteristics play a critical role [102]. In the context of cancer cells employing epigenetic mechanisms to develop resistant phenotypes, researchers showed that the combination of epigenetic drugs with conventional treatments can sensitize the cancer cell [103]. For instance, Vorinostat is proposed as a sensitizer of lung cancer cells to 5-FU by negative regulation of thymidylate synthase expression and enhancement of *p21waf1/cip1* expression via promoter histone acetylation [104].

**Figure 2:**
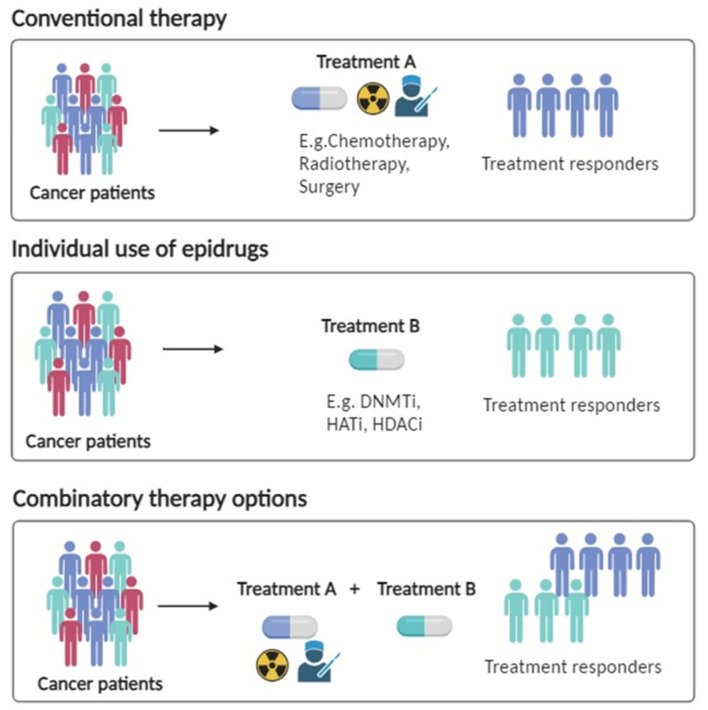
Graphical representation of how epidrugs can be employed either independently or in conjunction with other treatments, and emerging epigenome-editing technologies, such as DNMTi (DNA methyltransferase inhibitors) and HDACi (histone deacetylase inhibitors), offer additional options for cancer therapy.

Specific examples of epigenetic drugs used to increase the effect of standard therapies in lung cancer are given below (Figure 2).

### 6.1. Vorinostat

In one multi-institutional, open-label, phase I dose-escalation study in patients with EGFR-mutated (exon 19 deletion and L858R mutation) NSCLC and a BIM deletion polymorphism, Vorinostat was associated with gefitinib. The maximum tolerated dose (MTD) of Vorinostat was monitored, which was determined as the maximum dose level at which two or fewer of six patients showed dose-limiting toxicity (DLT). The authors used real-time qPCR to assess the effects of Vorinostat on the expression and splicing of BIM transcripts. They used BIM exon 2 expression as a surrogate expression marker for all BIM transcripts and assessed the expression of BIM exons 3 and 4, which reflect the BIM isoforms lacking the proapoptotic BH3 domain and having the proapoptotic BH3 domain, respectively. Treatment with Gefitinib and Vorinostat reduced the BIM mRNA exon 3/exon 4 ratio in all 11 individuals studied. Vorinostat, when combined with gefitinib, increased acetylated histone H3 protein expression, and decreased the BIM mRNA exon 3/exon 4 ratio in peripheral blood mononuclear cells (PBMC). In conclusion, they established that 400 mg biweekly Vorinostat paired with 250 mg daily Gefitinib is the optimal dose for phase II investigations in patients with NSCLC who are positive for both BIM deletion polymorphisms and EGFR mutations [105].

Another prospective, non-randomized, multicenter phase I/II trial was conducted to determine the maximum tolerated dose, toxicity profile, and efficacy of Erlotinib combined with Vorinostat. Patients with advanced NSCLC with EGFR mutations and worsening disease after at least 12 weeks on erlotinib were eligible. To assess the efficacy of the combination, the maximum tolerated dose of Vorinostat and Erlotinib was chosen as the recommended dose for phase II (RDP2). The major endpoint was the 12-week progression-free survival rate (PFSR 12 w). The phase I-II experiment enrolled 33 participants in total. Anemia, tiredness, and diarrhea were the most common toxicities among the 25 patients treated at the RDP2. On alternate weeks, a full dose of continuous Erlotinib with Vorinostat 400 mg p.o., QD can be safely delivered. However, the combination had no substantial efficacy in EGFR-mutated NSCLC patients [106].

In a different study, 52 patients with NSCLC, among which 22 had sensitive EGFR mutations, were enrolled and treated with 250 mg of Gefitinib and 400 mg of Vorinostat. The median PFS in 43 assessable patients in phase II was 3.2 months, with an overall survival (OS) of 19.0 months. There were 16 partial responses and six cases of disease stability. The response rate in EGFR-mutant NSCLC was 77%, the median PFS was 9.1 months, and the median OS was 24.1 months. Anorexia and diarrhea were the most common side effects. Treatment with 250 mg Gefitinib daily and 400 mg Vorinostat bimonthly was feasible and well tolerated. This combined dose did not increase PFS in an unselected patient sample. However, this combination seems to have the potential to improve gefitinib efficacy in EGFR-mutant NSCLC [107].

### 6.2. Panobinostat

Panobinostat is an HDAC (histone deacetylase) inhibitor and radiosensitizing agent that targets cancer epigenetics. The safety and efficacy of combining oral Panobinostat with radiation (RT) or chemoradiotherapy (CRT) in patients with inoperable stage III NSCLC were assessed in this phase I research. A parallel dosage-escalation approach was used in the trial, which combined oral Panobinostat twice a week (dose escalation 20, 30, 45 mg) with either palliative RT (group A) or radical CRT (group B) utilizing a normal chemotherapeutic combination of Cisplatin and Etoposide. In group A, disease control was 66%, PFS was 3 months, and median OS was 9 months. Because of infection-related problems, the Panobinostat dose in group B was not increased beyond 20 mg. Serious adverse events included opportunistic infection linked to treatment-related lymphopenia and febrile neutropenia with no known cause. All the patients had a partial response to treatment and were still alive after 33 months of follow-up, compared to a phase III study where patients were only treated with the same CRT and had a median overall survival of 21.5 months. Panobinostat is tolerated when paired with palliative-dose RT at doses of up to 45 mg twice a week. Dose-limiting toxicities inhibited Panobinostat dose escalation with CRT [108].

Panobinostat (LBH589), effectively inhibited the proliferation of NSCLC cell lines A549, Calu-1, H226, H460, H838, and SKMES-1 at IC50 concentrations ranging from 4 to 31 nmol/L through pleiotropic mechanisms that included crosstalk with EGFR signal transduction cascades. Combination therapy with Carboplatin effectively controlled anchorage-independent clonogenic survival to a significantly greater extent than either monotherapy. Carboplatin and Panobinostat administered at clinically relevant doses to NOD-SCID xenograft mice significantly slowed disease progression by 92% when compared to the negative control (*p* = 0.0026), which was greater than the 28% and 54% achieved with Carboplatin (*p* = 0.220) or Panobinostat (*p* = 0.017) alone. These findings show that Panobinostat has potent anti-NSCLC action and can chemosensitize tumors to Carboplatin, indicating that this combination method should be studied further in clinical trials [109].

### 6.3. Belinostat

As an alternative anticancer therapy, a novel combination of histone deacetylase inhibitor PXD101 (Belinostat) and CDK inhibitor CYC202 (Seliciclib) was studied. Combination therapy resulted in a significant reduction in cell proliferation compared to PXD101 alone in p53 wild-type A549 cells at therapeutically feasible concentrations of CYC202 (15 M). After concurrent therapy, there was a significant increase in apoptosis that occurred independently of cell cycle arrest. This finding was supported by higher levels of cleaved caspase-8, caspase-3, and PARP. The combined treatment increased the amounts of p53 and truncated BID proteins while decreasing the levels of Mcl-1 and XIAP proteins. Further apoptotic pathway research demonstrated that caspase inhibitors, but not p53 silencing, dramatically reduced cytotoxic increase. Furthermore, the improved efficacy of this combination was validated in p53 null H2444 cells, indicating the potential of this combination for the treatment of NSCLC that is resistant to the effects of standard p53-inducing drugs [110].

A synergistic combination consisting of cisplatin and Belinostat was reported in Pt(platinum)-resistant NSCLC cells. Belinostat was discovered to preferentially increase cisplatin-induced apoptosis in Pt-resistant cells. Cisplatin-resistant cells have an efflux transporter ABCC2 overexpression, an upregulation of the DNA repair gene ERCC1, and an increased DNA repair ability. Belinostat was discovered to suppress ABCC2 expression in Pt-resistant cells. HDAC inhibitors have been demonstrated to affect the expression of an estimated 4–12% of genes, owing to histone acetylation’s relaxation of DNA in the gene promoter and access to transcriptional regulators [111].

### 6.4. Trichostatin A (TSA)

In a comprehensive study, 81 lung cancer patients and 36 healthy and benign pulmonary lesion participants were assessed for the expression of insulin-like growth factor (IGF) binding protein-2 (IGFBP2). The expression was further validated in an additional cohort of 84 lung cancer patients, together with an evaluation of the prognostic and chemoresistant significance of IGFBP2. Remarkably, it was discovered that Trichostatin A (TSA), a well-known histone deacetylase (HDAC) inhibitor, effectively reversed chemoresistance in cell lines characterized by elevated IGFBP2 expression. The mean expression of IGFBP2 was considerably higher in lung cancer patients than in controls, and it increased as the cancer progressed to an advanced stage. Furthermore, high IGFBP2 expression was found to be an independent predictor of chemoresistance; over-expressed IGFBP2 increases cell activity, and TSA can counteract the chemoresistance caused by high IGFBP2 expression by increasing autophagy. Furthermore, after adjusting for stage, histology, EGFR mutation, age, smoking, and surgery, multivariate analysis revealed that lung cancer patients with greater blood IGFBP2 had a worse survival result, with a hazard ratio of 8.22 (95% CI 1.78–37.92, *p* = 0.007) [112].

The bicellular tight junction molecule claudin-2 (CLDN-2) is extensively expressed in lung cancer tissues and promotes adenocarcinoma cell growth. In human lung adenocarcinoma cells, downregulation of the tricellular tight junction molecule angulin-1/LSR causes malignancy via EGF-dependent CLDN-2 and TGF-dependent cellular metabolism. Human lung adenocarcinoma A549 cells and normal lung epithelial cells were treated with Trichostatin A (TSA) and Quisinostat (JNJ-2648158) with or without TGF to investigate the detailed mechanisms of antitumor activities of HDAC inhibitors in lung adenocarcinoma. Both HDAC inhibitors enhanced anguin-1/LSR, decreased CLDN-2, accelerated G1 arrest, and blocked A549 cell migration. Furthermore, TSA, but not Quisinostat, with or without TGF-induced cellular metabolism, as determined by the oxygen consumption rate, showed mitochondrial respiration. In 2D culture, TSA and Quisinostat boosted LSR and CLDN-2 expression while decreasing CLDN-1 expression in normal human lung epithelial cells.

In 2D culture, Quisinostat, but not TSA with TGF, enhanced CLDN-7 expression. Both HDAC inhibitors reduced epithelial barrier disruption as evaluated by FD-4 permeability generated by TGF in 2.5D culture. TSA and Quisinostat have the potential to be used in the treatment of lung adenocarcinoma by altering the expression of angulin-1/LSR and CLDN-2 [113].

### 6.5. Decitabine and 5-Azacytidine

Significantly reduced levels of CTGF (connective tissue growth factor) mRNA and protein were identified in NSCLC cell lines (A549 and Calu-1) as well as in lung malignant tissues obtained from a cohort of 98 NSCLC patients. These findings suggest also that CTGF expression in NSCLC can be epigenetically controlled. CTGF mRNA and protein levels were dramatically elevated in A549 and Calu-1 cells after incubation with 5-Aza-2′-deoxycytidine (5-dAzaC) or TSA. The normal human bronchial epithelial cell line, Beas-2B, exhibited the same effect. Application of tested drugs (5-dAzaC and TSA) could be beneficial in NSCLC for at least two reasons: they not only enhanced the amount of CTGF transcripts and protein in NSCLC cell lines, but they also led to cell proliferation reduction. Because A549 cells have wild-type TP53 and both alleles of TP53 are eliminated in Calu-1 cells, they found that both 5-dAzaC and TSA could inhibit NSCLC cell proliferation in a p53-independent way. The authors demonstrated that 5-dAzaC at doses of 10 and 15 µM could decrease A549 and Calu-1 cell multiplication without significantly reducing cell viability [114].

Immune-deficient models were used to investigate the efficiency of sequential 5-Azacytidine (5-Aza) combined with HDACi in vivo. When 5-Aza and ITF-2357 were used, the authors saw a significant reduction in tumor burden across three xenograft models, two for established NSCLC cell lines and one for a KRAS, KDR, TP53 mutant primary patient-derived xenograft (PDX) model. At the doses utilized in the study, the combination of 5-Aza and MS-275 had no meaningful effectiveness in the H460 xenograft model, the most sensitive cell line evaluated. Aza and MGCD0103 had great efficacy in H460 cells but only limited efficacy in PDX. One of the most optimal strategies involved low-dose 1-week 5-Aza followed by 1-week ITF-2357 in two mouse models of NSCLC. For three months, mice were given a combined medication treatment. While mock-treated animals acquired massive adenocarcinoma lesions in the lungs, combination epigenetic treatment prevented the development of these macroscopic lesions and resulted in a 60% reduction in tumor area in the treated mice [67].

## 7. Conclusions

Studies on the epigenetics of lung cancer underscore the promising potential of epidrugs as a novel approach to lung cancer therapy. Clinical trials employing both single- and multi-drug therapies, as well as immunotherapy, have shown encouraging results, offering the prospect of increased life expectancy and reduced chemotherapy-related side effects for patients. However, it is evident that a comprehensive understanding of the mechanisms underlying the action of epidrugs in these therapies remains a critical area of investigation. To bridge this knowledge gap, cellular and molecular assays complemented by translational feedback from clinical reports have proven to be invaluable tools. These assays serve to elucidate and associate epigenetic modifications with the modulation of phenotypes, shedding light on the intricate interplay between epigenetics and lung cancer.

In order to effectively utilize epigenetic therapy, it is crucial to find predictive indicators when new trials become available for enrollment. The current level of comprehension regarding the mechanisms and rationales underlying the functioning of these agents is basic and necessitates further development. The examination of alterations in gene expression resulting from pharmacodynamic changes can yield valuable information regarding the subsequent impacts of these agents. Additionally, such investigations have the potential to identify predictive indicators that could be of utility in the context of epigenetic therapies. The intersection of cancer epigenetics and cancer immunology, notably immune checkpoint inhibitors, holds great promise for enhancing cancer therapy efficacy [115]. However, the translation of preclinical findings to clinical practice necessitates addressing multifaceted challenges. Recognizing the intricate nature of epigenetic regulations, including the potential for compensation and context-dependent effects, is crucial. Furthermore, the potential of combining immunotherapy with epigenetic modulators, specifically DNMTi and HDACi, is highly encouraging. The combination of these therapeutic agents has shown the capacity to regulate the immune response and surmount acquired resistance to immunotherapy, although the exact mechanisms behind these effects have yet to be fully understood [116].

Moreover, the complex interplay between PRMT dysregulation, particularly in PRMT5, and MTAP loss in NSCLC underscores the need for extensive research, validating the pursuit of PRMT5 and MAT2A inhibitors as promising therapeutic avenues.

As research in this field continues to advance, the integration of epigenetic insights into clinical practice holds the potential of more effective, targeted, and personalized approaches to combatting lung cancer, ultimately benefiting patients and improving their quality of life.

## Figures and Tables

**Table 2:** Clinical Trials Investigating DNMT Inhibitors in Lung Cancer Treatment.

Study ID, Type and Phase	Objective	Patient Characteristics and Condition	Treatment	Outcome	Status
ID: NCT00978250 Interventional Phase II Study	Assess the synergy of FdCyd and THU in controlling tumor growth and evaluate their combined safety and tolerability	25 Patients aged 18 to ≥65 years, Non-Small Cell Cancer	DNMT inhibition with 5-Fluoro-2′-Deoxycytidine (FdCyd) + Tetrahydrouridine (THU)	Median progression-free survival for the group of 25 participants was 2.3 months, with a 95% confidence interval ranging from 1.6 months to 3.9 months	Completed
ID: NCT05960773 Interventional Phase II Study	Evaluation of DNA demethylating agent, in individuals with BAP1 Cancer Predisposition Syndrome who have subclinical or early-stage mesothelioma	Estimated number of patients: 15 Age ≥ 18 years.	Decitabine/Cedazuridine	Pre-recruitment phase	Not yet recruiting Patients (estimated study completion 2026)
ID: NCT01207726 Interventional Phase II Study	Impact of 5-azacitidine and Entinostat on 3-year progression-free survival in stage I non-small cell lung cancer patients after resection	13 Patients aged 18 to ≥65 years	5-Azacitidine and Entinostat	Study terminated prematurely due to low enrollment, making data analysis and conclusions impossible	Terminated
ID: NCT02664181 Interventional, Phase II Study	Effectiveness of combining the investigational drug tetrahydrouridine-decitabine (THU-Dec) with nivolumab, compared to nivolumab alone, in patients with Non-Small Cell Lung Cancer (NSCLC)	13 patients, Age ≥ 18 years	Tetrahydrouridine/Decitabine + Nivolumab	A total of 50% of the participants experience progressive disease. A total of 25% showed stable disease. A total of 25% presented partial remission.	Ongoing
ID: NCT00385398 Interventional, Phase II Study	Evaluate the impact of stereotactic radiosurgery (SRS), temozolomide, and erlotinib hydrochloride on cognitive function in NSCLC patients with brain metastases. Determine the frequency of O6-methylguanine-DNA methyltransferase promoter methylation in these patients.	Patients with Age ≥ 18 years	Erlotinib hydrochloride, Temozolomide, Radiation	Specific data or reasons for the study’s withdrawal were not provided	Withdrawn
ID: NCT02959437 Interventional Phase I Phase II	The study consists of two parts, with Part 1 dedicated to dose escalation for safety assessment. Following dose determination, Part 2 enrolls subjects with previously treated NSCLC, microsatellite-stable colorectal cancer (CRC), head and neck squamous cell carcinoma, urothelial carcinoma, and melanoma into expansion cohorts.	70 Patients, Age ≥ 18 years	Azacitidine Pembrolizumab Epacadostat	All participants received a minimum of one dose of the investigational drug and had evaluable baseline and on-treatment biopsies. None of the individuals were enrolled in Part 2 of the study due to the early termination of the research as a strategic decision.	Terminated

**Table 3:** Phase 3 Clinical Trials Investigating HDAC Inhibitors in Lung Cancer.

Study ID, Type and Phase	Objective	Patient Characteristics and Condition	Treatment	Outcome	Status
ID: NCT00005093 Interventional Phase III	Evaluate the efficacy and safety of gemcitabine, both as a standalone treatment and in combination with CI-994, in patients with advanced non-small cell lung cancer	Age ≥ 18 years No additional data regarding patient accrual is provided	Tacedinaline CI-994 Gemcitabine hydrochloride	No outcome data available provided	Completed
ID: NCT00128102 Interventional Phase III	Assessing the oral investigational drug vorinostat’s efficacy and safety versus a placebo in treating advanced malignant pleural mesothelioma after prior chemotherapy, with the primary goal of enhancing overall survival	A total of 661 patients, Age ≥ 18 years with confirmed pleural mesothelioma	Vorinostat Placebo	In the Vorinostat group, the objective response rate (ORR) stands at 0.63% with a 95% Confidence Interval (CI) ranging from 0.08% to 2.25%. Conversely, the placebo group reports an ORR of 0.31% with a 95% CI spanning from 0.01% to 1.71%	Completed
ID: NCT00473889 Interventional Phase III	Assess the survival outcomes of advanced non-small cell lung cancer patients treated with vorinostat in combination with paclitaxel and carboplatin	253 participants, Age ≥ 18 years, confirmed with NSCLC	Vorinostat Paclitaxel Carboplatin Placebo	Out of 125 participants, 28 (22.4%) responded favorably to Vorinostat, Paclitaxel, and Carboplatin treatment, while 97 (77.6%) showed an unfavorable disease response. In the placebo group with Paclitaxel + Carboplatin, 36 (28.8%) exhibited a positive response, with 87 (69.6%) demonstrating an inadequate disease response	Terminated based on the recommendation by the DSMB following a pre-planned protocol interim analysis because the endpoint was not achieved
